# Changes in qualitative and quantitative traits of birch (*Betula pendula*) pollen allergenic proteins in relation to the pollution contamination

**DOI:** 10.1007/s11356-021-13483-8

**Published:** 2021-03-25

**Authors:** Monika Ziemianin, Jacek Waga, Ewa Czarnobilska, Dorota Myszkowska

**Affiliations:** 1 Department of Clinical and Environmental Allergology, Jagiellonian University Medical College, Botaniczna 3, 31-503 Kraków, Poland; 2 Department of Plant Breeding, Physiology, and Seed Science, University of Agriculture in Kraków, Podłużna 3, 30-239 Kraków, Poland

**Keywords:** Birch pollen, Allergenic proteins, Pollen seasons, SDS-PAGE, Particulate matter, Kraków

## Abstract

Birch (*Betula pendula*) pollen causes inhalant allergy in about 20% of human population in Europe, most of which is sensitive to the main birch allergen, Bet v1. The aim of the study was to find out (i) whether and how the analysed birch individuals differ in regard to composition of individual subunits of pollen proteins and to protein content in these subunits; (ii) whether the level of particulate matter relates to concentration of Bet v1 allergen. Study was performed in Southern Poland, in 2017–2019. Pollen material was collected at 20 sites, of highly or less polluted areas. Protein composition was analysed by SDS-PAGE, while the concentration of Bet v1 was evaluated by ELISA. The obtained results were estimated at the background of the particulate matter (PM10) level and the birch pollen seasons in Kraków. The electrophoregrams of pollen samples collected at different sites showed huge differences in staining intensities of individual protein subunits, also among important birch allergens: Bet v1, Bet v2, Bet v6 and Bet v7. The level of Bet v1 was significantly higher in the pollen samples collected at the more polluted sites. While the birch pollen allergenic potential is determined, the both pollen exposure and the content of the main allergenic components should be considered, as factors causing immunological response and clinical symptoms manifestation in sensitive individuals.

## Introduction

Currently, the problem of the atmospheric pollution concerns up to 96% of the European Union urban population, according to the latest report of European Environment Agency (Air Quality in Europe 2020; https://www.eea.europa.eu). The anthropogenic pollutants play a major role as etiological factors of many inflammatory, allergic and irritant diseases (Anderson et al. 2013; Hernandez and Peden 2014; Senechal et al. 2015). Epidemiological studies performed in Europe indicated a higher percentage of people with pollen allergy in urban areas than in places with a low level of the abiotic pollution (Behrendt and Becker 2001; Samoliński et al. 2009; World Health Organization 2018; Air Quality in Europe 2020).

The pollutant particles can be regarded as pollen allergy-facilitating agents or as a cause of allergy symptoms including pollen allergy (*pollen allergy-initiating*) (Senechal et al. 2015). They are treated as ‘adjuvants,’ enhancing the immunoreactivity of plant proteins and affecting indirectly the intensity of symptoms in pollen allergy-prone patients related to the overproduction of sIgE antibodies. This phenomenon, recognised and described for almost 20 years (Ring et al. 2001; Bousquet et al. 2001; Baldacci et al. 2015), was confirmed by a number of studies conducted during the pollen seasons, e.g., Feo Brito et al. (2007) and Guilbert et al. (2018).

Anthropogenic pollutants influencing the biological and reproductive functions of pollen are treated as bio-indicators of pollution impact (Iannotti et al. 2000). Particles of particulate matter (fractions PM2.5 and PM10) including metals (nickel Ni, cadmium Cd, arsenic As and lead Pb) and gaseous contaminants, like sulphur dioxide (SO_2_), carbon oxide (CO), nitrogen dioxide (NO_2_) and ozone (O_3_), may be transported on the surface of pollen grains and may modify the morphological structure of exine and molecular structure of proteins (Chehregani et al. 2004; Lu et al. 2014; Mousavi et al. 2019; Visez et al. 2020), strengthening their immunoreactivity indirectly (Armentia et al. 2002; Suárez-Cervera et al. 2008; Cuinica et al. 2014; Schiavoni et al. 2017, and citations therein). It is stated that atmospheric PM affects more people than any other pollutants, exacerbating asthma symptoms, including life-threatening attacks (Kim et al. 2015; Phosri et al. 2017). While particles with a diameter of 10 microns or less can penetrate and lodge deep inside the lungs, the even more health-damaging particles of 2.5 microns or less can penetrate the lung barrier and enter the blood system.

The problem of a long term existing plants in the polluted air refers especially to the perennial plants, including the allergenic trees. In central and northern Europe, the main source of tree pollen is the Fagales order, including the Betulaceae family (Beck et al. 2016) among which birch allergens are the most allergenic (Skjoth et al. 2013). The silver birch (*Betula pendula*) is considered as a model plant for the study of pollen-patient reactions. Birch trees produce a large amount of pollen (5.5 million pollen grains per male inflorescence); their pollen seasons are short, dense, but intensive (Myszkowska 2013; Kubik-Komar et al. 2019).

Nowadays, 6.4–22.4% of the European population is sensitive to birch pollen, half of which manifest clinical symptoms, including allergic rhinitis (AR), accompanied by asthma symptoms and oral allergy symptoms (Pfaar et al. 2017; Biedermann et al. 2019). Research on allergenic proteins' physicochemical properties using SDS-PAGE electrophoresis showed that birch pollen proteins form a complex composed on dozen to tens subunits and fractions. Bet v1, the main birch allergen, belongs to the pathogenesis-related proteins (PR-10 subfamily) and is a diagnostic marker for identifying patients with genuine (initial) sensitisation to birch pollen and also can be responsible for birch pollen-related plant-food allergy (OAS) (Breiteneder and Kleine-Tebbe 2016).

Bet v1 recognises sIgE in 95% of patients, while the other components are recognised as follows: Bet v2 (10–38%), Bet v3 (10%), Bet v4 (20%), Bet v5 (35%) and Bet v7 (up to 20%) (Moverare et al. 2002). Variation in the patients reactivity to the different birch pollen components is related to the individual sensitivity of the immune system, which can be modified regarding the features of birch specimen, being modified by the different environmental conditions (Sedghy et al. 2018).

Kraków (southern Poland), treated as a highly polluted city, is the right place for the study on relationship between natural allergens and pollution. Our previous analyses singled out that the permissible level of PM10, estimated according to the Polish recommendations (permissible level: 50 μg/m^3^, information level: 100 μg/m^3^, alert level: 300 μg/m^3^) (http://monitoring.krakow.pios.gov.pl/standardy-jakosci-powietrza/last assessed 20.11.2020), is more than twice exceeded from October to April (Ziemianin et al. 2016). The comparative study of tree pollen seasons and the concentration of PM10 showed also the frequent co-occurrence of high birch pollen concentration (>155 pollen/m^3^) related to the risk of asthma dyspnea and PM10 concentration >50 μg/m^3^, in March–April (Ziemianin et al. 2016). The potential impact of a multiply exposure was confirmed by experimental study performed in 26 persons (12 patients with birch pollen allergy and 14 healthy controls). In allergic patients, the percentage of basophils activated in vitro using Basophil Activation Test (BAT) was 6.46% higher after the simultaneous activation of dust and pollen compared to the activation with dust and birch pollen alone (Czarnobilska et al. 2020). Considering the presence of AR in 30% of children and 25% of adolescents in Kraków (Czarnobilska and Mazur 2016), we have undertaken the study on the indirect impact of PM10 particles on trees occurring in the urban area, based on the own epidemiological and experimental studies and the literature reports.

Data presented above and general knowledge about pathogenesis of birch allergy allow us to hypothesise that content and composition of birch pollen proteins play also an important role in birch pollen allergenicity control. In this study, we analysed composition of birch pollen protein subunits by SDS-PAGE. Moreover, the subunits of 35, 28, 18, 17 and 14 kDa were chosen for detailed quantitative analysis for evaluation protein content in these subunits. In our opinion, this may be an important factor influencing birch pollen protein allergenicity. The literature data allow to expect that these proteins are indeed the pollen allergens: Bet v6, Bet v8, Bet v7, Bet v1 and Bet v2, respectively (Martijn et al. 2009).

The aim of the study was to find out (i) whether and how the analysed birch individuals differ in regard to composition of individual subunits of pollen proteins and to protein content in these subunits; (ii) whether the level of particulate matter relates to concentration of Bet v1 allergen.

## Materials and methods

### Localisation of birch sites

**Fig. 1:**
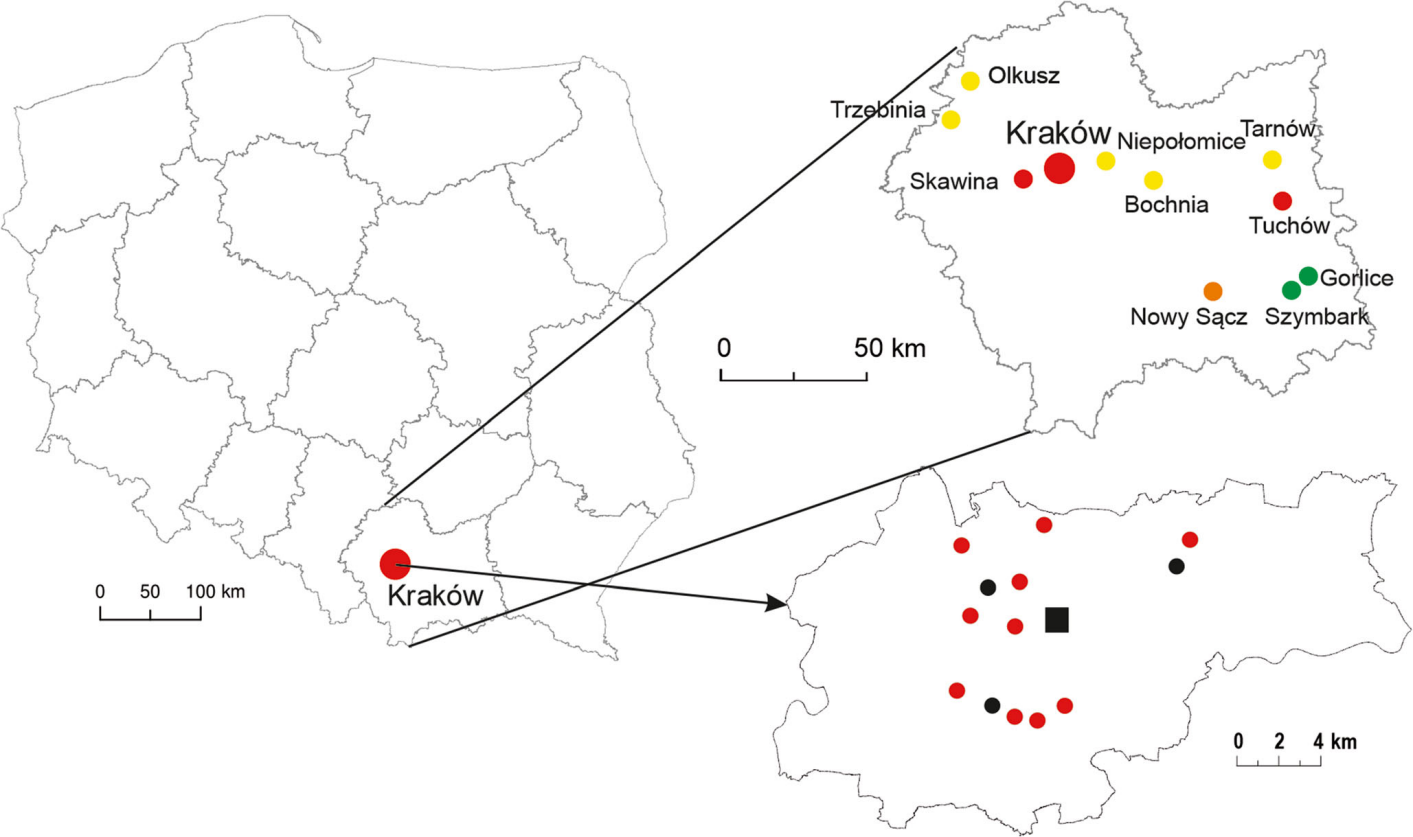
Study sites, including birch stands in Kraków and in the other localities out of Kraków (Małopolska province). Dots’ colour corresponds to the categorised level of PM10 as described in Material and methods chapter (green and yellow dots refer to the lower values [SIn1–SIn2], and orange and red dots refer to the higher values [SIn3–SIn4]). Sites in Kraków (red dots) were grouped near the three pollution monitoring stations (black dots). The stationary volumetric pollen sampler was marked as a red square

Ten sites in Kraków and 10 sites outside of the city (Lesser Poland) were chosen to collect birch pollen samples (Fig. 1). ‘Outside’ sites are located up to 135 km away from Kraków. The selection criterion for studied sites in Kraków was the distance of 9.5 km from the point of the volumetric measurement (downtown) and the location of the three selected pollution stations (Fig. 1). The sites outside of Kraków were classified on the basis of the annual and monthly pollution levels and occurrence birch trees in the vicinity of these stations. The minimum distance of the station from the birch site is 50 m (in Olkusz), while the maximum distance is 850 m (in Skawina). Most of the sites in Malopolska province were located in the city centre, in housing estates, often near cemeteries, but away from motorways, except at Bochnia (near the heavy traffic road between Tarnów and Kraków).

### Pollen and pollution data

The volumetric measurement of pollen concentrations in Kraków was performed Hirst type devise (Hirst 1952) located in the city centre, at the height of 20 m a.g.l. Sample collection was described in our previous papers (e.g., Myszkowska 2013, 2014). The research methodology consists of performing microscopic slides with material taken from the air, as well as scanning and counting pollen grains and fungal spores using a light microscope (400 × magnification). Pollen grains were counted along 4 longitudinal transects. The pollen concentration was given in 1 m^3^ of air/24 h. All procedures were performed in accordance with the recommendations of the European Aerobiology Society (EAS) (Galan et al. 2014, 2017). The measurements were carried out in March–May, in every studied year (2017–2019), and the special 24-h head has been installed in the stationary sampler, in order to be up to date with daily data. The season characteristics, including season start, end, duration and Annual Pollen Index (APIn) were calculated according to the recommendation of EAS (Galan et al. 2017).

PM10 data were obtained from the open database of Malopolska Inspectorate for Environmental Protection in Krakow (http://monitoring.krakow.pios.gov.pl/last assessed; 15/03/2020). For the study purposes, the PM10 data were collected from 13 monitoring stations in 2010–2019 to categorise study sites according to the exposure level, using a 'quantitative and qualitative' method based on the ordinal scale. Firstly, the lowest and highest mean monthly values calculated for each station (including four stations in Kraków) were selected. The obtained ranges were divided into four equal subintervals (SIn), which were numbered from 1 to 4 [SIn1–SIn4], from the lowest to the highest values, respectively). Each monitoring station in a given month was assigned un appropriate SIn relating to the mean monthly PM10 value. Then the mean SIn value was calculated for each station, wherein stations with mean value less than or equal to 2 were (SIn1–SIn2) designated as low pollution sites, while above 2 (SIn3–SIn4) as high polluted sites.

### Pollen collection and storage

The catkins collection and allergen measurements have been done according to methodology reported by Buters et al. (2008) (in own modification). Since the middle of March, phenological observations of birch specimens were carried out to determine the appropriate time of catkins collection, which means time before the flowers were fully opened. At the turn of March and April, male catkins of birches were collected at all selected sites, from the same three specimens/site (60 per year), about 50 catkins per specimen, during rainless days. The catkins were stored in the paper envelopes and dried for at least 5 days. Inflorescences not fully opened were put into a glasshouse at 13% humidity, 28 °C for 1 day. The birch pollen was then sieved, weighed and stored in polypropylene vials at –30 °C.

### Electrophoresis

Birch pollen proteins were extracted according to Helander et al. (1997) procedure with several modifications. Hundred mg of each pollen sample were incubated with 400 μl of 0.1-M acidic ammonium carbonate (NH_4_HCO_3_) solution in 1.5-ml Eppendorf plastic tubes with gently shaking, overnight. The obtained suspension was centrifuged (1,000 rpm, 10 min). The capacity of 50 μl of each protein extract was added to 100 μl of the protein sample buffer composed of 6-M urea and 2.5% SDS with addition of 1.5% mercaptoethanol to reduce disulphide bonds. The extracted pollen proteins diluted in the buffer described above were shacked on Vortex, centrifuged again and immediately applied for electrophoretic separation.

Birch pollen proteins were separated by the SDS-PAGE in the Mini Protean II Cell electrophoretic chamber using the 4–10% ‘ready to use’ gradient gel (Mini Protean TGX Precast Gels) in the Tris-HCl buffer using tenfold diluted manufacturing concentrate. All components used for electrophoretic analysis (i.e., electrophoretic chambers, power supplier, gels and buffer concentrate) were from Bio-Rad, USA. Five microlitres of each protein extract were loaded to the wells formed in the gel slabs. The central well of each slab was loaded with Protein Precision Plus molecular weight marker (10–250 kDa) (Bio-Rad, USA). The proteins were separated for 45 min at constant voltage 200 V. The current values changed from 5 to 30 mA during the run. After electrophoresis gels were stained overnight in a ComassieBrillant Blue (R250 + G250), these were destained in distilled water for 24 h. Each of obtained electrophoregrams was recorded using digital camera Lumix FZ 1000 as JPEG files. Gel images were processing using FastStone Image Viewer 7.5. Only crop tool and brightness/contrast scroll bars were applied equally across the entire images for improving their visual properties. Cropped and uncropped gels were labelled by arrows, clamps, asterisks and numbers in MS Power Point.

Molecular weights of separated subunits and fractions of birch pollen proteins were calculated using free software ‘Gel Analyser.’ The evaluation of protein content in individual protein bands was performed using Image J free software for Windows. This application changes electrophoretic bands into peaks and calculates the areas under the peaks, simulating densitometric measurements. The obtained densitometric data were expressed in relative units (RUs) and accepted as the relative protein content for individual subunits of separated birch pollen proteins.

### Evaluation of electrophoretic/densitometric data

For the evaluation of differences in protein content among five considered subunits, the following statistical indicators were used:

1. Mean protein content (MPC) calculated for individual protein bands. This indicator was calculated based on densitometric measurements and expressed in RUs. The obtained MPC values show how much protein is contained in each of the five SDS-PAGE subunits observed in eighteen birch individuals.
2. Coefficient of variability (CV%) calculated for the obtained MPC values. This indicator informs to what extent the protein content in individual protein bands is a stable trait in consecutive years and in different environments.
3. Range of CV% differentiation within groups of analysed objects (RCV).
4. Number of individuals of the lowest CV% values (<10%) within groups of analysed objects (NCV).

The MPC values were calculated (i) for five individual protein bands (MPC 1) as average value of eighteen birch specimen (five groups); (ii) for individual birch trees (MPC 2) expressed as average value of five considered protein bands for each of eighteen considered birch individuals (18 groups).

RCV allows to evaluate stability of MPC within groups, while NCV confirms reliability of the conclusion based on the analysis of quantitative differentiation. Mean MPC 1, RCV 1 and NCV 1 inform about allergenic potential of individual protein subunits while MPC 2, RCV 2 and NCV 2 inform about allergenic potential of considered birch individuals.

The results of quantitative analysis were classified as profitable, unprofitable and intermediate. The obtained results were accepted as profitable when the objects within groups were characterised by low mean MPC values, the narrow range of RCV and the high NCV number. On the contrary, high mean MPC values in combination with the narrow range of RCV and the low NCV number were accepted as unprofitable results.

### Measurements of Bet v1 concentration in pollen

Before the immunoenzymatic measurements of Bet v1 concentration, the birch pollen collected from 60 specimen/year was extracted at 20-mg pollen/4 ml in 0.1-M NH_4_ HCO_3_, pH 8.1, in 15-ml polypropylene tubes, after 1-min vortexing and 4 h in an endover-end rotator at 100 rpm at room temperature in the dark. The mixture was transferred to 2-ml Eppendorf vials, centrifuged for 5 min at 13,000 g, and the supernatant was collected. To 400-μl extract, BSA was added to make 0.1% w/v, and the samples were lyophilised at 30 °C and stored at 4 °C until analysis. Bet v1 allergen concentration was measured using the enzyme-linked immunosorbent assay (ELISA) immunoassay with the monoclonal antibodies (ELISA kit for Bet v1, Indoor Biotechnologies). Five concentrations of Bet v1 were calibrated to each plate using the Bet v1 standard reagent. The lyophilisate samples were dissolved in 2 ml of 1% BSA PBS-Tween buffer and then analysed in three dilutions (100×, 200× and 400×) in triplets. In the first stage, the plate was coated with the monoclonal antibody (4B10) and left overnight at 4 °C. Then the manufacturer instructions were followed, among others, by adding a biotinyled antibody (2B10). Bet v1 concentration was expressed as ng/10 mg of pollen.

### Statistical analyses

Basic descriptive statistics (mean, minimum, maximum, standard deviation and CV%) were used to describe the dynamics of the pollen seasons and to present quantitively the variability of proteins separated by electrophoresis. Bet v1 concentration was studied using multivariate analysis of variance (ANOVA for factorial designs). Detailed comparisons among the groups identified in the analysis of variance were performed using contrast analysis. All analyses were performed using Statistica 13.

## Results

### Pollen seasons in Kraków, in 2017–2019

**Fig. 2:**
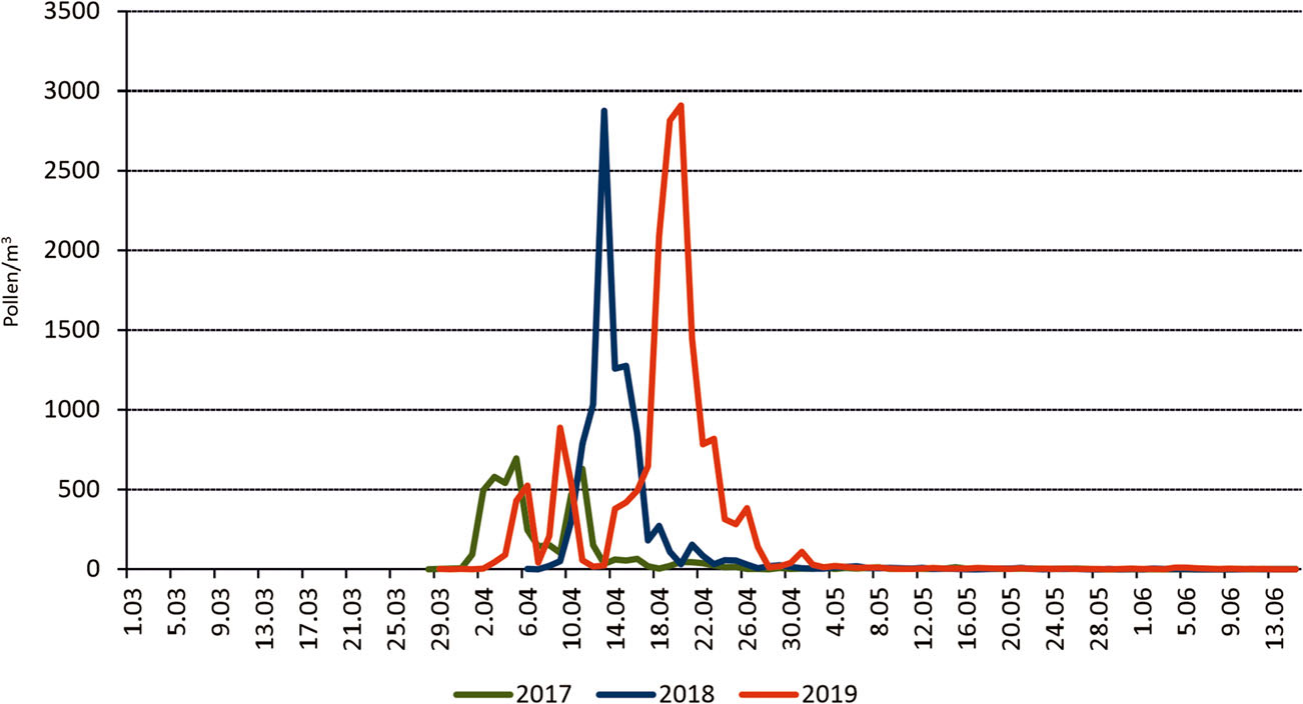
Seasonal dynamics of the daily birch pollen concentrations in Kraków, in 2017–2019

Seasonal dynamics of the birch pollen occurrence in 2017–2019 in Kraków showed the clear differences in time and intensity of the pollen seasons. The earliest pollen season started in 2017, followed by 2019 and finally by 2018. In 2017 and 2019, two peaks of pollen concentrations have been seen clearly, although in 2017, their intensity was definitely lower (Fig. 2).

Descriptive statistics indicated that among the pollen season characteristics in 2017-2019, the most deviating from the retrospective long-term data (1991-2016), was the season end, which occurred earlier in all study seasons in relation to the previous 26 years. On the other hand, the Annual Pollen Index (APIn) was definitely several times higher in 2019 in comparison with the previous seasons (1991-2016) (Table 1). The intensity of the pollen season in 2019 is reflected also by the highest number of days with the daily concentration above 75 pollen/m^3^.

**Table 1:** Birch pollen characteristics in 2017–2019 in Kraków in relation to the descriptive statistics calculated for 1991–2016

Season	Season start^a^	Season end^a^	Season duration^b^	APIn^c^	Maximum concentration	Date of max conc.	Days with pollen/m^3^ > 75^b^
2017	92^d^	114^d^	23	4,623	697	95^d^	12
2018	100	114^d^	15^d^	9,291	2,877^d^	103	12
2019	95	117^d^	23	16,649^d^	2,911^d^	110	21^d^
	101.58	129.27	28.69	4,933.65	961.26	108.38	11.46
Min	88.00	114.00	16.00	833.00	119.00	93.00	2.00
Max	114.00	190.00	77.00	19,791.00	4,199.00	123.00	24.00
SD	6.77	13.93	12.84	4,905.17	981.62	7.35	6.46
95% CI	98.84; 104.31	123.64; 134.90	23.51; 33.81	2,952.41; 6.914.89	564.77; 1.357.74	105.41; 111.36	8.85; 14.07
*V%*	6.66	10.78	44.74	99.42	102.12	6.79	56.39

^a^Season characteristics calculated as the consecutive day of the year from the 1st of January

^b^Number of days

^c^Annual pollen index calculated for 95% method — season start calculated as the first day when the concentration reaches 2.5% of annual total; season end calculated as the last day with the concentration reaching 97.5% of annual total

^d^Data out of 95% CI

$\overline{x}$, arithmetic mean; *Min*, minimum value; *Max*, maximum value; *SD*, standard deviation; 95% *CI*, confidence interval, *V%*, coefficient of variation

### Electrophoretic patterns of birch pollen proteins

**Fig. 3:**
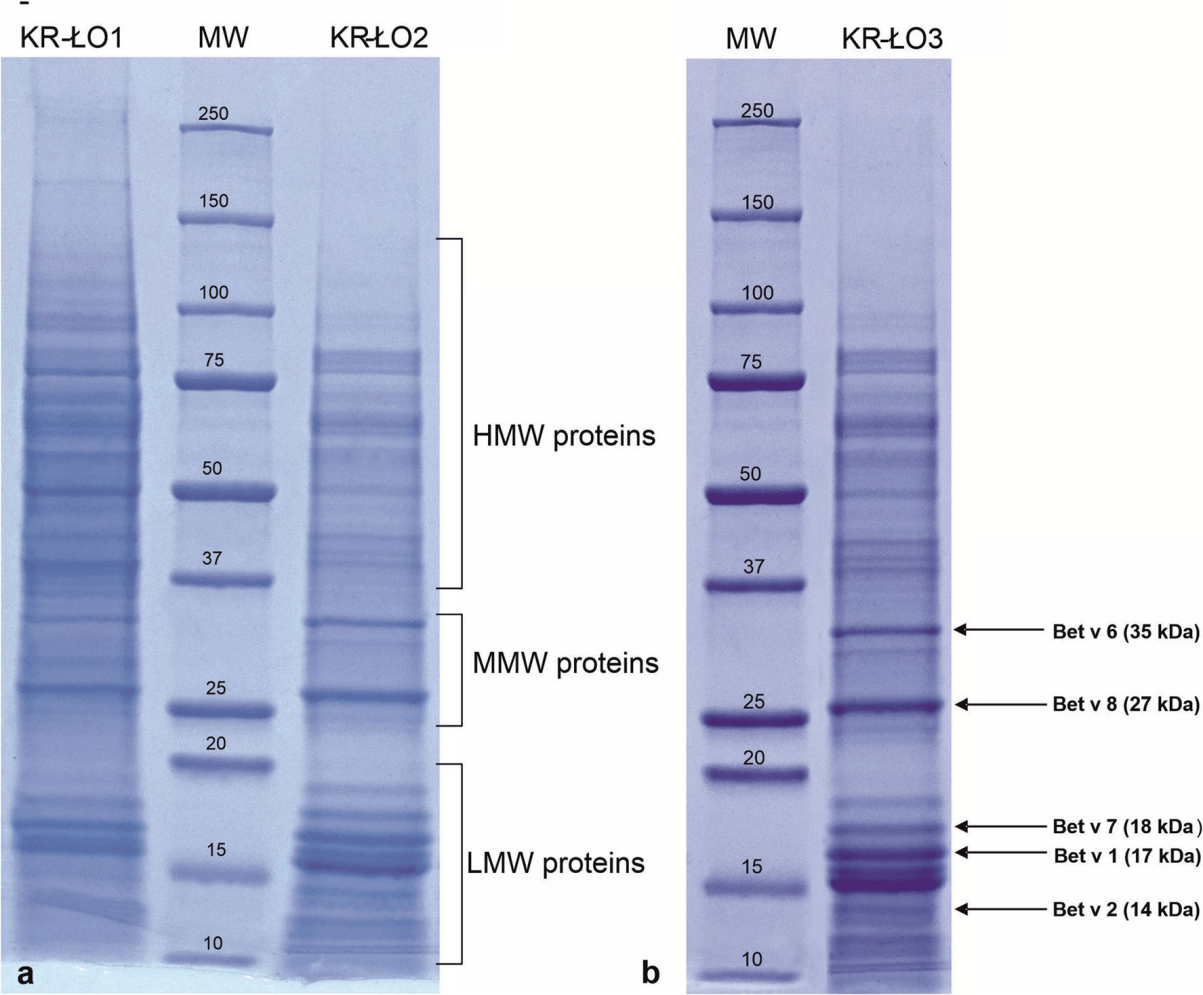
General characteristic of birch pollen proteins based on the SDS-PAGE electrophoregrams performed in pollen samples collected from birch site in Kraków: (**a**) distribution of the protein complex into three zones of high molecular weight (HMW), medium molecular weights (MMW) and low molecular weights proteins (LMW) marked in brackets; (**b**) proteins of MW: 35, 27, 18, 17 and 14 kDa occurred in most of analysed pollen samples and probably related to the main birch allergens marked by the arrows

Electrophoretic separations under SDS-PAGE conditions showed that birch pollen proteins were composed of dozen to tens subunits and fractions depending on analysed birch individuals. Their molecular weights (MWs) range from several to over hundred kDa. The specificity of the obtained separations allows to divide the set of electrophoretic bands into three groups differentiated in regard of their MW: high (HMW), medium (MMW) and low (LMW) of proteins (Fig. 3a).

Two protein bands of MWs 35 and 27 kDa observed in the MMW zone and three proteins of MWs 18, 17 and 14 kDa in the LMW zone are related to the most important birch pollen allergens Bet v6, Bet v8, Bet v7, Bet v1 and Bet v2, respectively (Fig. 3b).

#### Qualitative differences

**Fig. 4:**
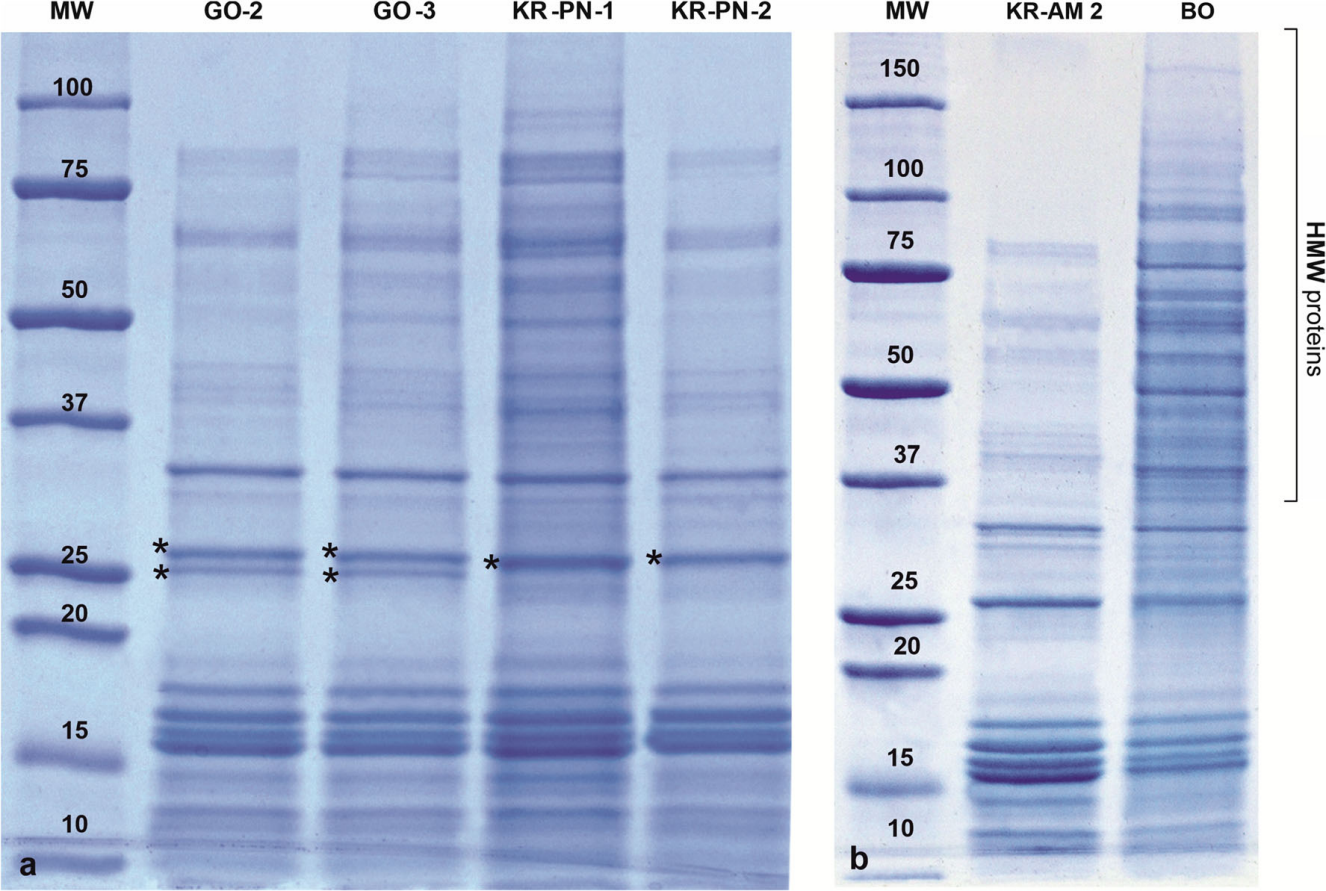
Qualitative and quantitative differences observed among analysed birch pollen samples: (**a**) individual protein band of molecular weight 28 kDa present in samples 16 (KR-PN-1) and 17 (KR-PN-2) segregate into a pair of closely localised proteins in samples 14 (GO-2) and 15 (GO-3) (marked by asterix) as an example of qualitative differences in protein composition; (**b**) SDS-PAGE of two birch pollen samples: KR-AM 2 and BO illustrating a strong decreasing of staining intensity in HMW proteins zone and disappearance of protein bands over 80 kDa as quantitative differences in protein content among separated birch pollen proteins in different birch individuals

The analysed pollen samples showed only a small number of qualitative differences consisting in changes of some protein band combinations. The observed differences were two-fold in character. First, some individual bands segregated into two bands, like the 28-kDa protein in the samples GO 2 and GO 3 vs. KR-PN 1 and KR-PN 2 collected in 2019 (Fig. 4a). In several other cases, individual bands slightly change their MW and — in consequence — their localisation on the electrophoregrams.

#### Quantitative differences

Most of the differences among pollen proteins were quantitative in character. They were expressed as changes of the staining intensity of corresponding protein bands identified in different birch individuals. A huge differences could be observed while comparing electrophoregrams of pollen samples collected in the same year but in different sites like, e.g., KR-AM 2 vs BO (Fig. 4b).

Analysis of the MPC values for five groups differing in regard of individual protein bands (each one of groups comprising eighteen birch trees) showed the lowest MPC 1 values for 18 kDa (potentially Bet v7 – 839 RU) and 14 kDa (potentially Bet v2 – 970 RU) proteins. Analysis of mean protein content (MPC) in five considered protein bands showed the lowest MPC values for 18 kDa and 14 kDa proteins, the highest for 28 kDa, while the intermediate values were calculated for 35 kDa and 17 kDa proteins (Table 2). As to RCV 1 indicators, three groups (for proteins of: 28, 18 and 17 kDa) show narrow, one group (14 kDa) wide and one group (35 kDa) intermediate range of CV% differentiation. In turn, two groups (28 and 17 kDa) show high number, one group (14 kDa) — low number and one group intermediate number of birch individuals of CV% values not exceeding 10%. No one of analysed groups differentiated in regard to MW of chosen subunits showed profitable values for all of three statistical parameters considered all together. However, parameters for the group 18 kDa (Bet v7) could be considered as close to profitable.

**Table 2:** Mean values of densitometric measurements and coefficients of variability for chosen birch pollen samples

No	Birch individual	35 kDa (Bet v6)		28 kDa (Bet v8)		18 kDa (Bet v7)		17 kDa (Bet v1)		14 kDa (Bet v2)		Mean MPC 2	RCV 2*	NCV 2
		MPC	CV (%)	MPC	CV(%)	MPC	CV (%)	MPC	CV (%)	MPC	CV (%)			
1	KR-AM-2	1,108	28	1,733	11	842	29	2,726	10	341	34	**1,350**^**c**^	11–34 (23)^b^	2^b^
2	KR-RE-1	1,226	33	1,612	12	585	37	1,944	2	1,651	3	**1,404**^**c**^	2–37(35)^b^	3^b^
3	KR-RE-3	1,205	26	2,010	17	775	23	1,029	16	934	50	**1,191**^**c**^	16–50 (34)^b^	0^a^
4	KR-NH-1	2,211	21	2,727	10	1,186	5	2,060	9	2,040	51	2,045^a^	5–51 (46)^b^	3^b^
5	KR-NH-2	1,368	3	2,389	7	774	25	1,466	8	634	38	**1,326**^**c**^	3–38 (35)^b^	3^b^
6	KR-NH-3	2,029	1	2,371	4	972	22	2,175	4	1,467	29	1,803^a^	**1–29 (28)**^**c**^	3^b^
7	KR-GR-1	2,022	19	2,089	7	687	12	1,507	5	1,472	4	1,555^b^	**4–19 (15)**^**c**^	**4**^**c**^
8	KR-GR-2	1,588	18	2,508	2	1,006	29	1,398	14	1,026	35	1,505^b^	2–35 (33)^b^	2^b^
9	KR-GR-3	1,223	1	2,588	4	1,277	42	2,240	4	361	1	1,538^b^	1–42 (41)^b^	**4**^**c**^
10	KR-DA-1	2,081	13	2,138	3	791	5	1,555	10	131	89	**1,339**^**c**^	3–89 (86)^a^	**4**^**c**^
11	KR-DA-3	1,962	2	2,199	11	797	18	1,928	9	248	32	**1,427**^**c**^	**2–32 (30)**^**c**^	3^b^
12	KR-BE-1	516	7	1,224	16	800	1	2,141	38	1,289	31	**1,194**^**c**^	1–38 (32)^b^	2^b^
13	KR-BE-2	999	71	2,444	30	xxx	xxx	1,308	2	1,452	99	1,551^b^	2–99 (97)^a^	1^a^
14	GO-1	1,464	56	2,279	24	699	13	1,689	31	1,409	101	1,508^b^	13–101 (88)^a^	1^a^
15	GO-2	1,889	24	2,487	11	734	28	1,336	23	433	31	**1,376**^**c**^	11–31 (20)^b^	1^a^
16	OL-1	1,424	39	2,699	12	868	30	1,917	18	884	25	1,558^b^	18–39 (21)^b^	1^a^
17	OL-2	1,607	18	2,469	11	906	5	1,704	15	811	40	1,499^b^	5–40 (35)^b^	3^b^
18	SK-2	1,652	67	2,498	47	567	32	1,473	18	882	1	**1,414**^**c**^	1–67 (66)^a^	1^a^
Mean MPC 1		1,532^b^		2,248^a^		**839**^**c**^		1,755^b^		**970**^**c**^				
RCV 1*			1**–**71 (70)^b^		**2–47 (45)**^**c**^		**1–42 (41)**^**c**^		**2–38 (36)**^**c**^		1–101 (100)^a^			
NCV 1			6^b^		**13**^**c**^		6^b^		**12**^**c**^		4^a^			

*MPC*, mean protein content (expressed in relative values calculated for densitometric measurements coming from 3 years of investigations); *CV*%, coefficients of variability for given MPCs; *Mean MPC* 1, mean values of MPC for individual protein subunits; *Mean MPC 2*, mean values of MPC for individual birch trees; *RCV 1*, range of CV% differentiation predicted for five individual protein subunits; *RCV 2*, range of CV% differentiation predicted for 18 individual birch trees; RCV 1 and 2*, differences between the highest and the lowest RCV values are given in the brackets; *NCV 1*, number of trees of CV% values not exceeded 10% for individual protein subunits; *NCV 2*, number of protein subunits of CV% values not exceeded 10% for individual birch trees

^a^Unprofitable values of considered traits

^b^Intermediate values of considered traits

^c^Profitable values of considered traits (in bold)

Statistical indicators for 18 groups of birch objects indicated almost the linear variability of densitometric parameters. Nine out of 18 birch individuals were accepted as objects of the low, seven as medium and two as high content of allergenic proteins based on MPC 2 values (Table 2). In turn, 10 out of 18 birch individuals showed the narrow, four medium and four wide range of CV% values differentiation (based on RCV 2 values). As to the NCV 2 indicator, three birch individuals showed high, nine medium and six low numbers of allergenic proteins of CV% values not exceeding 10%. No one birch individual showed profitable values for all of three statistical parameters considered all together. However, parameters of four individuals KR-AM 2, KR-RE 1, KR-DA 3 and KR-BE 2 could be considered as close to profitable.

### Bet v1 concentration in pollen

**Fig. 5:**
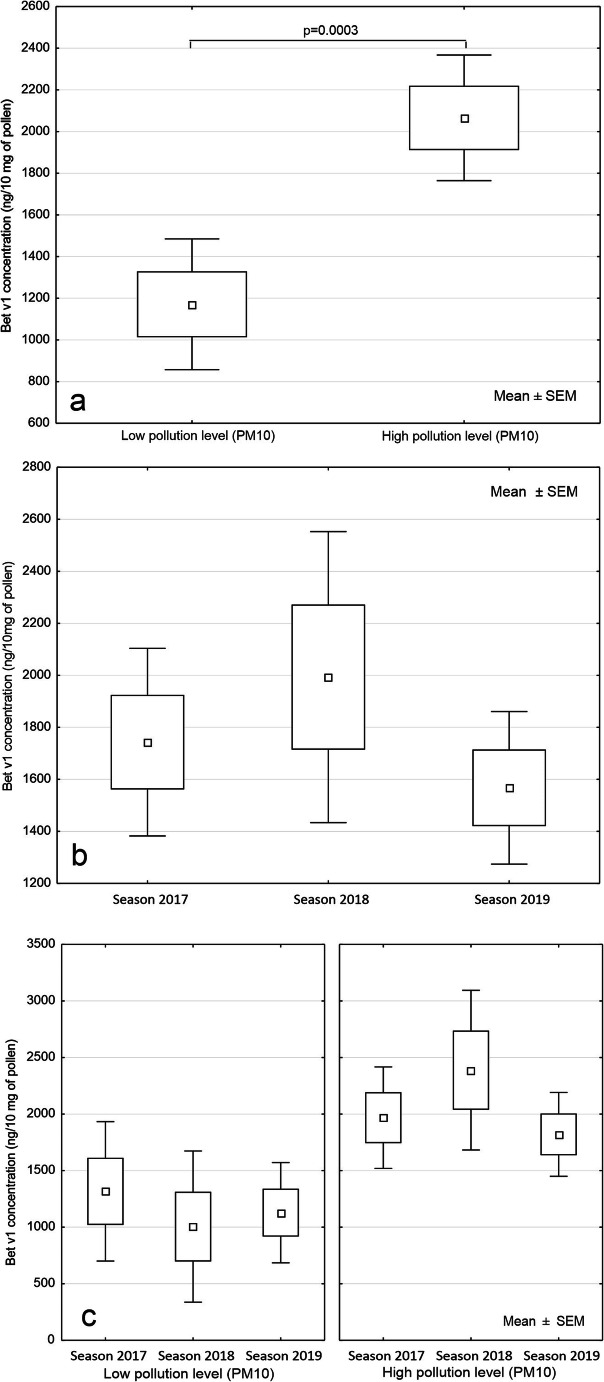
Bet v1 concentration in all studied years in relation to (**a**) the pollution level and (**b**) the year of the study, regardless the pollution level **c** both factors: the year of the study and pollution level.

The obtained Bet v1 concentrations were analysed using multivariate ANOVA. Two factors the pollution level (PM 10) (higher vs. lower) and the studied seasons (2017, 2018 and 2019) allowed to distinguish six groups of assessed relations. ANOVA for factorial designs indicated the statistically significant differences in Bet v1 concentrations (F(5;129) = 3.39; *p* = 0.0065) among the studied groups. One-dimensional tests indicated the level of PM10 as a factor significantly related to the mean Bet v1 concentration in pollen (F(5;129) = 13.81; *p* = 0.0003) (Fig. 5a). The mean Bet v1 concentration was statistically higher in pollen samples collected at the more polluted sites, reaching, on average, more than 2.000 ng/10 mg of pollen while in the specimen growing at the less contaminated sites; the mean Bet v1 concentration was almost twice as low as for more polluted sites.

The year of observations did not influence the Bet v1 level in a statistically significant way (F(5;129) = 0.29; *p* = 0.7500). Slight differences were noted between Bet v1 concentrations in 2017 and 2018, while in 2019, the lowest Bet v1 values were obtained (Fig. 5b). Generally, the widest range of Bet v1 concentration was observed in 2018.

Discussing both factors influencing the Bet v1 concentration, it is clearly seen that the scheme of mean Bet v1 values differs in the consecutive years (Fig. 5c). The highest Bet v1 concentration was obtained at more polluted sites in 2018, while at less polluted sites, this year was found as less contaminated by Bet v1. Results of a multivariate ANOVA confirmed that an important factor affecting the Bet v1 level is PM10 concentration; the contrast analysis showed that only in 2018, the relation between PM10 and Bet v1 was statistically significant (Table 3).

**Table 3:** The results of contrast analyses

Factor 1 (season)	Factor 2 (pollution level)	*t*	*p*
All	High/low	3.72	0.000
2017	High/low	1.69	0.094
2018	High/low	−3.06	0.003
2019	High/low	−1.61	0.109

## Discussion

AR provoked by pollen allergens is one of the most frequent allergic diseases, especially in highly industrial European countries (Pfaar et al. 2017; Biedermann et al. 2019). It is stated that the main cause of this problem is the simultaneous effect of pollen exposure and air pollution on allergenic symptoms, including the direct impact of allergenic particles within the pollen seasons and changes in the allergenic protein content resulted from a long-term abiotic stress (Bédard et al. 2019).

Birch pollen seasons are treated as rather stable in relation to the season timing, in comparison, e.g., to the early pollinating trees (Myszkowska 2013; Myszkowska and Majewska 2014; Malkiewicz et al. 2016; Pfaar et al. 2017; Kubik-Komar et al. 2019), although in Western central and Northern Europe, the pollen seasons of these three taxa overlap partially (Biedermann et al. 2019). Their season stability in Kraków has been already reported, and the current analysis confirmed the results of the previous studies (Myszkowska 2013, 2014; Myszkowska and Majewska 2014). The results a multisite study performed in 2001–2016 in Poland indicated that the season end is more variable than the season start (Kubik-Komar et al. 2019), but this characteristic is not as important as the season onset, due to the *priming effect* (Frenz 2001; Pfaar et al. 2017) related to the clinical symptoms. People sensitive to birch pollen are warned of being threatened by extending exposure of pollen, which corresponds to the Bet v1 release (Buters et al. 2012).

A biannual rhythm of the birch pollen season intensity since 2006 (alternating seasons with high and low APIns) reported by pollen monitoring stations in Poznań (Grewling et al. 2012) and in Gdańsk (Latałowa et al. 2002) was confirmed in Kraków, if the long data series were considered (Kubik-Komar et al. 2019). In 2016, the APIn was twice as high as in 2019, which can disrupt a bit the natural, biannual rhythm, but in 2018, the index value was higher than in 2017. It is worthy underlying that in 2019, the number of days with pollen concentration exceeding 75 pollen/m^3^ and making a threat for sensitive people was almost twice as high as in the previous seasons. Considering the relative stability of the birch pollen seasons and the a natural rhythm of pollen occurrence, it is easier to predict the increase in the pollen occurrence and to find the potential relationship between pollen and pollution in the air.

Birch pollen proteins analysed during the three studied pollen seasons showed qualitative and quantitative differentiations. Qualitative traits controlled by individual genes are independent on environmental conditions, while quantitative properties (i.e., protein content in individual subunits) are under control of numerous genes and strongly depend on environmental changes of trees habitats (Wright 1978). In our current study, qualitative modifications of protein electrophoretic profiles were observed, in the protein bands originating from pollen from more polluted (Kraków, Piaski Nowe — KR-PN) and low polluted site (Gorlice — GO). These modifications may be caused by spontaneously occurring mutations that generate a range of new allelic variants (Hedrick 1985) and may possibly influence the proteins allergenicity. While investigating *Lilium martagon* pollen grains by SDS-PAGE after DEP exposure, some new bands similarly as in our studies appeared. Moreover, immunoblotting studies indicated that new bands, not allergenic in the wild pollen type, strongly reacted with human anti-IgE (Hedrick 1985). The authors concluded that pollution can carry pollen allergen molecules, induce new proteins (allergens) and also act as adjuvant for allergens.

Comparison of electrophoregrams of two pollen samples collected from central of Kraków (KR-AM 2) of high PM10 level and from Bochnia (BO) of lower PM10 level are a good example illustrating how particulate matter can influence quantitative traits. One possible biochemical mechanism explaining modifications of proteins by air pollutants is that in polluted, urban areas, pollen grains are exposed to a complex of environmental elements, which may act as mutagens causing discrete, point mutations changing the gene sequences and gene expression, what in consequence influence their protein and allergen content (Bryce et al. 2010; Schiavoni et al. 2017). The expression of allergenic molecules in pollen grains is attributable to adaptation of plants to abiotic stress (Schiavoni et al. 2017).^.^ Chehregani et al. (2004) analysing the SDS-PAGE protein profiles of different pollen samples from *Zinnia elegans* collected in Tehran (Iran) at polluted and nonpolluted sites found the same bands in polluted and nonpolluted pollen grains, but in all polluted pollen grains, the protein content decreased in response to air pollution.

Mousavi et al. (2019) reported the year-to-year differences in the expression of some of *Ailanthus altissima* pollen proteins, especially in the basic region of 2-DE separations, identifying five allergenic proteins. Electropherograms obtained by Mousavi et al. (2019) contained two protein spots that were present in both extracts but were able to stimulate the human immune system and specific IgE recognition. The authors underlined that the implementation of allergomic tools for the safety assessment of newly introduced and invasive plant species would help in the comprehensive monitoring of proteomic and transcriptomic alterations involving environmental allergens.

In our studies, the most profitable for human health, the lowest MPC 1 values, were found for 18- and 14-kDa protein subunits (putative Bet v7 and Bet v2, respectively). Both of these proteins are related to the cross-reactivity rather (cyclophilin and profilin), then the allergens responsible for the systematic reaction (Moverare et al. 2002). Unfortunately, unprofitable values of RCV 1 and NCV 1 — in case of the 14-kDa subunit — allow to expect the high instability of the protein content in succeeding years and variable environmental conditions. This strong instability can result in non-expected allergic reactions in the sensitive individuals. Such an effect was reported by Shahali et al. (2009), who compared the structure and the allergenic protein content in Arizona cypress (*Cupressus arizonica*) pollen collected just after microsporangia dehiscence and 2 weeks later in urban areas. After that time, numerous cracks and collapses appeared in pollen surfaces. Moreover, western blotting revealed that sera-specific immunoglobulin E of all allergic subjects reacted with a new allergen of 35-kDa MW, while the decrease in reaction to the band at approximately 45 kDa (Cup a 1, the main *C. arizonica* allergen) appeared.

In the case of the 17-kDa protein subunit (putative Bet v1), profitable (from biological point of view) RCV 1 and NCV 1 values cause that intermediate (but close to unprofitable) mean MPC 1 value becomes an unprofitable trait from birch allergy point of view. Both RCV 1 and NCV 1 indicators cause the high stability of the intermediate protein content in the 17-kDa protein subunit. When structural traits of this subunit also favour strong allergenicity of the 17-kDa subunit, the stability of protein content may cause negative effects on human health in the long time period. The fact that Bet v1 is the main birch pollen allergen able to sensitise more than 90% of birch allergic people (Moverare et al. 2002) suggests correctness of the hypothesis mentioned above and makes an element of novelty of this research. Possibility of monitoring the allergenicity of both individual protein subunits and birch individuals in combination with simplicity of experimental design of the research enforces the value of presented strategy as a possible, useful diagnostic tool in practical allergology.

The results of Bet v1 analysis in pollen indicate a slight variability in main birch allergen concentration, regarding any of the studied years, which is worthy assessed in relation to the pollen season intensity. Buters et al. (2008) found that the release of Bet v1 from pollen was more than 5 times higher in 2003 than in 2002 in two regions in Germany, whereas the allergen concentration was 3 times higher in the part of lower pollen occurrence. Our results are compatible with the German observations, because the lowest Bet v1 concentration was received in 2019, when the birch pollen season was the most intensive. It indicates the inversely proportional relation (the more intensive pollen release, the less content of Bet v1), but it should be confirmed by the longer observations. It can be stressed that the mean Bet v1 concentration (around 5.000 ng/10 mg of pollen) obtained in German studies in the more polluted region was slightly higher than in our study. The study on Pla a 1 concentration (the main allergenic protein of *Platanus hybrida* pollen), performed in two urban localities in Spain, showed also the clear annual differences, which were inversely proportional to the total pollen protein biomass (Antunes et al. 2020).

Analysing the relationship between Bet v1 concentration and the PM10 level, it should be stressed that the generative organs of birches are formed in mature individuals, usually after the flowering period (May–September) of the preceding year. At the first stage (morphogenesis), the morphological elements of flowers are formed, followed by micro- and microsporogenesis, initiated around the middle of August, when the generative cells are created. The process is inhibited in November, when the winter dormancy starts (lasting up to February, March or even April, depending on the year). Genetic factors definitely determine the formulation of pollen and the proteins therein; however, different environmental factors have an effect on the amount of protein, like in case of Bet v1 (Ahlholm et al. 1998). For these reasons, PM10 dominating among abiotic pollutions, from October to April, should be considered as a factor, which may partially affect the formation of pollen and the protein components. During winter, when the PM concentration is the highest, the suspended dust can settle on inflorescences, and hypothetically, when the pollen release starts, PM particles can be adhered on pollen surface and induce allergen release and agglomeration of pollen material, loaded with allergens by diffusion of proteins (Chehregani et al. 2004; Visez et al. 2020). Such pollen–particle complexes, forming in the polluted environments, can affect allergy and asthma in allergenic individuals (Schiavoni et al. 2017).

Although in our study, the higher Bet v1 concentration was related to the higher PM10, we must be aware that plants try to respond suitably to pollution by adjusting their metabolism (minimum damage done due to air pollution induces flavonoids accumulation to significantly higher levels in polluted pollen than in controlled ones). Plant protective response may include an increase in antioxidant enzymes and metabolites and induction of protection-related secondary metabolite genes especially flavonoids (Rezanejad 2009; Galveias et al. 2021). Therefore, not only the pollen and aeroallergens’ concentration in the air suggested by many authors (Buters et al. 2012; Plaza et al. 2020) should be considered while exposure reliably estimation but also the features of allergenic proteins, like Bet v1 released from pollen, developing in polluted areas.

## Conclusions

Environmental conditions, including particulate matter, PM10, strongly modify birch pollen protein characteristics in different ways. Protein content calculated for individual subunits and fractions of electrophoretically separated birch protein complex was highly differentiated depending on birch individuals and pollution level. Only in some of the analysed birch trees, the protein content in particular bands was a highly stable trait, while in the others, it was strongly dependent on environmental conditions. Variability or stability of the protein content in particular subunits and fractions containing allergenic proteins may affect the allergenic potential of birch specimen. The concentration of Bet v1, the stress protein, is significantly higher in areas of the high air pollution level, in comparison with that of the low air pollution level, probably in response to the PM10 level, as one of the abiotic stress conditions. While the birch pollen allergenic potential is determined, the both pollen exposure and the content of the main allergenic components should be considered, as factors casing immunological response and clinical symptoms manifestation in sensitive individuals.

## Data Availability

Pollen data, electrophoresis pictures and Bet v1 concentrations measured in frame of the study were stored as project documentation by the National Science Centre. PM10 data were obtained from the open database of the Malopolska Inspectorate for Environmental Protection in Krakow (http://monitoring.krakow.pios.gov.pl). The datasets used and analysed during the current study are available from the corresponding author on reasonable request.
